# Thyroid Disorders in Patients with Polycystic Ovarian Syndrome in a Tertiary Care Center: An Observational study

**DOI:** 10.31729/jnma.8833

**Published:** 2024-12-31

**Authors:** Jyotshna Sharma, Bimita Mahat, Sanjeeb Tiwari, Niraj Kumar Singh, Ranjana Yadav, Durga Thapa

**Affiliations:** 1 Department of Obstetrics and Gynaecology, Kathmandu Medical College, Sinamangal, Kathmandu, Nepal; 2 Department of General Practice, Tribhuvan University Teaching Hospital, Maharajgunj, Kathmandu, Nepal; 3 Department of Internal Medicine, Narayani Hospital, Birgunj, Parsa, Nepal

**Keywords:** *oligomenorrhea*, *pcos*, *thyroid gland*

## Abstract

**Introduction:**

Polycystic ovarian syndrome is the most common endocrine and metabolic disorder in women of childbearing age, affecting 3-15% of women worldwide, leading to reproductive, metabolic, and psychological issues. Patients with polycystic ovarian syndrome require rigorous thyroid function detection, monitoring, and correction over time. In this study, we aimed to evaluate the clinical presentations and thyroid dysfunction in patients with polycystic ovarian syndrome.

**Methods:**

An observational study was done in patients with polycystic ovarian disease presenting to a tertiary care centre over six months, from December 2023 to May 2024 Total population sampling was done. All the women during the study period diagnosed with polycystic ovarian disease based on Rotterdam criteria were included in the study after getting ethical approval from the institutional review board. (Reference number: 20102023/03).

**Results:**

The mean age of women in the study was 24.74±5.01 years. A total of 28 (31.46%) patients of polycystic ovarian disease had hyperthyriodism, hypothyroidism was found in 13 (14.60%) and subclinical hypothyroidism was found in 6 (6.74%) patients. All the women had menstrual irregularities. Regarding androgenic characteristics, 53 (59.55%) of patients presented with hirsutism, 43 (48.51%) presented with acne, 13 (14.61%) women had alopecia, and 4 (4.49%) women had acanthosis.

**Conclusions:**

Hyperthyroidism, hypothyroidism and subclinical hypothyroidism were prevalent in polycystic ovarian disease patients, emphasizing the need for thorough thyroid evaluation in polycystic ovarian disease patients due to its impact on metabolic and reproductive health. High rates of menstrual irregularities, androgenic symptoms like hirsutism and acne, and fertility challenges were also prevalent, aligning with findings from similar studies.

## INTRODUCTION

Polycystic ovarian syndrome (PCOS) is a common condition in women characterized by hyperandrogenism, ovulatory dysfunction (including menstrual disturbance), and polycystic ovarian morphology (PCOM).^1^ It is the most common endocrine and metabolic disorder in women of childbearing age, affecting 3-15% of women worldwide, leading to reproductive, metabolic, and psychological issues that can severely impact their physical and mental health.^2^ In the presence of thyroid disorders, ovarian morphology changes to polycystic. Hence, thyroid abnormalities are one of the exclusion factors before making a diagnosis of PCOS in any woman.^3^ The clinical symptoms of PCOS are directly impacted by thyroid function, which makes the clinical PCOS phenotype more diverse. Therefore, patients with PCOS require rigorous thyroid function detection, monitoring, and correction over time, which will mitigate or perhaps fully prevent the further deterioration of PCOS symptoms.^2^

A previous study from Nepal has reported a prevalence of 22.86% positive thyroid peroxidase antibody in a patient with PCOS.^4^ This study has however not looked into the clinical presentation of the patients. Therefore, in this study we aimed to evaluate the clinical presentations and thyroid dysfunction in PCOS patients.

## METHODS

An observational cross-section study was conducted at Kathmandu Medical College and Teaching Hospital, Kathmandu, Nepal, following ethical approval from the institutional review committee (Reference number: 20102023/03). Patients with polycystic ovarian syndrome (PCOS) visiting the hospital over six months, from December 2023 to May 2024 were enrolled in the study. Total population sampling was done. All the women during the study period diagnosed with PCOS based on Rotterdam criteria^4^ were included in the study. The exclusion criteria were hyperprolactinemia, congenital adrenal hyperplasia, and virilising tumour. Data collection was carried out using a semi-structured questionnaire. The first half of the questionnaire included sociodemographic characteristics. The second half included serum levels of different hormones. Detailed clinical history, elaborate clinical examination, and laboratory investigations like blood glucose, thyroid stimulating hormone (TSH), and free thyroxine levels (free T3 and free T4) were performed in the PCOS population. The definition of normal for the thyroid tests was defined by institutional reference values: (1) free T3: 1.58-3.91 pg/ml, (2) free T4: 0.70 1.48 ng/dl, and (3) TSH: 0.35-4.94 mIU/ml. Thyroid function was evaluated by measurement of fasting serum thyroid stimulating hormone (TSH) and free thyroxine levels (free T3 and free T4). A transabdominal pelvic USG was performed to detect the presence of cystic ovaries. According to Rotterdam criteria, polycystic ovarian syndrome (PCOS) is defined by the presence of two of three of the following criteria: oligo-anovulation, hyperandrogenism, and polycystic ovaries (≥ 12 follicles measuring 2-9 mm in diameter and/or an ovarian volume > 10 mL in at least one ovary).^5^ According to World Health Organization (WHO) standards, a BMI of less than 18.5 kg/m^2^ was considered underweight, a BMI of 18.5 to 24.9 kg/m^2^ was considered normal weight, a BMI of 25 to 29.9 kg/m^2^ was considered overweight, and a BMI of 30 kg/m^2^ or more was considered obese. Class I (BMI 3034.9 kg/m^2^), class II (BMI 35-39.9 kg/m^2^), and class III (BMI >40 kg/m^2^) are further classifications of obesity.^6^ The collected data were verified for completeness and organized for subsequent analysis in Microsoft Excel. Statistical analysis was carried out using SPSS, employing descriptive statistics such as mean, median, frequency, standard deviation (SD), interquartile range (IQR) and percentage.

## RESULTS

There were 89 cases of PCOS within 6 months. The mean age of women was 24.74±5.01 years, 46 (51.69%) of the women were unmarried and 62 (69.66%) were parity, nulliparous (Table 1).

**Table 1:** Sociodemographic profile of PCOS patients (n=89).

Age group (years)	n (%)
Less than 20	14 (15.73)
21-30	65 (73.03)
31-40	8 (8.99)
More than 40	2 (2.25)
**Marital Status**	
Married	43 (48.31)
Unmarried	46 (51.69)
**Parity**	
Nulliparous	62 (69.66)
Primiparous	16 (17.98)
Multiparous	11 (12.36)

PCOS = Polycystic Ovary Syndrome

The mean BMI of women presenting with PCOS was 23.71±3.80. A total of 51 (57.30%) of women had normal BMI, 6 (6.74%) were underweight, 29 (32.58) were overweight and 3 (3.37%) were in obesity 1 category. Our study demonstrated that among the total PCOS patients, 47 (52.81%) patients had a thyroid disorder. Among total PCOS, 28 (31.46%)had hyperthyroidism, 13 (14.60%) had hypothyroidism, and 6 (6.74%) had subclinical hypothyroidism.

In our study, all women had menstrual abnormalities. A total of 79 (88.76%) women had oligomenorrhea and regarding androgenic characteristics, 53 (59.55%) of patients presented with hirsutism (Table 2). Among the total PCOS patients, 15 (16.85%) had complaints of subfertility and in 63 (70.78%) of the women PCOS was seen in ultrasound.

**Table 2:** Clinical Manifestations in PCOS (n=89).

Menstrual abnormalities	n (%)
Oligomenorrhea	79 (88.76)
Secondary amenorrhea	10 (11.24)
Dysmenorrhea	14 (15.73)
**Other clinical manisfestations**	**n (%)**
Hirsutism	53 (59.55)
Acne	43 (48.31)
Subfertility	15 (16.85)
Alopecia	13 (14.61)
Acanthosis	4 (4.49)

## DISCUSSION

In our study, the mean age of women with PCOS was 24.74±5.01 years. It is consistent with another study done in the United States of America that showed the mean age at diagnosis of PCOS was 26.9 years.^7^ Similarly, the majority of the women in our study belonged in the age group of 21-30 years. This differs significantly from a prospective study done in India where the majority of women with PCOS (78%) belonged to the age group of 13-19 years.^8^ This might be because this study included women in the age range of 13-25 years, but our study included women of all age groups diagnosed with PCOS. PCOS typically manifests around menarche, though some women may experience symptoms later. These days, PCOS is more commonly identified in early teens and might appear before menarche.^9^

Regarding the thyroid function, in our study, 31.46% of patients with polycystic ovary syndrome (PCOS) had hyperthyriodism; hypothyroidism was found in 14.60% of study subjects; and 6.74% of study subjects had subclinical hypothyroidism. In a study done in Nepal by Vaidya et al., hypothyroidism was seen in 21% of the total population.^10^ In a study done by Pervin et al., primary hypothyroidism was seen in 16.0% of patients which was the predominant thyroid abnormality; subclinical hypothyroidism was found in 9.60% of patients and primary hyperthyroidism was found in 8.0% of study patients.^11^ The unusually high prevalence of hyperthyroidism in your study compared to typical findings in the literature is indeed striking. This discrepancy may be influenced by several factors, such as regional differences, genetic predispositions, dietary factors (e.g., iodine intake), or even specific environmental exposures that might uniquely affect the study population.

The mean BMI of women presenting with PCOS was 23.71±3.80 in our study. In our study, the majority 57.30% of women had a normal BMI, 32.58% of women were overweight, 6.74% were underweight, and 3.3% of women were grade 1 obese. Another similar study done in Nepal by Vaidya et al. reported similar findings where the majority of the patients 56% had a normal BMI, 30% were overweight, and 11% were obese.^10^ However, another study by Mohapatra et al. reported 75.5% of total women with PCOS were obese.^12^ The higher percentage of obese patients might be due to their categorization of overweight and obese patients as obese PCOS patients. The majority of women among pregnant with PCOS in our study 69.66% were nulliparous. This high percentage is consistent with the fact that PCOS often leads to ovulatory dysfunction and infertility, making conception more challenging for affected women.

In PCOS, excessive androgen production hinders follicle maturation, leading to anovulation and conception difficulties due to the absence of a fully mature ovum.^13^ Patients also presented with various androgenic characteristics. A total of 59.55% of patients presented with hirsutism, and 48.51% of patients presented with acne in our study. In a study done in Bangladesh by Pervin et al. among PCOS patients, clinical hirsuitism was found in 58% study subjects, 16% of subjects had acne in their face.^11^ This discrepancy in presentation of acne may be influenced by several factors, including genetic, environmental, and lifestyle differences between populations. In our study, menstrual abnormalities were present in all the patients of PCOS. All women with PCOS in our study had menstrual abnormalities. Other studies have also reported higher percentages of menstrual irregularities in female suffering from PCOS. A study by Joshi et al. reported menstrual irregularities as the most common presenting complaint of PCOS patients which was seen in 83% of total female.^14^ Similarly, In a study conducted in China 76% of PCOS patients had an abnormal cycle.^15^ These findings suggest that menstrual irregularities, such as long or irregular cycles, are not only prevalent but also serve as a major clinical indicator for PCOS across diverse populations. In our study, USG features of PCOS was seen in 70.78% of the women. It is consistent with the study done by Najem et al., where USG features of PCOS was seen in 74% of total population.^16^ Although not every woman with PCOS may exhibit these ovarian features on ultrasound, this diagnostic method continues to play a central role in identifying the condition, especially in cases where clinical symptoms may vary.

Being a single-center-based study, the study results cannot be generalized to a broader population. Antithyroid peroxidase (anti-TPO) antibody measurement is absent in our study. Without anti-TPO data, it is challenging to determine whether cases of hypothyroidism are autoimmune in origin, which is relevant because autoimmune thyroid disease is often associated with PCOS and may influence thyroid management strategies. Additionally, the lack of anti- TPO data limits the ability to explore the full spectrum of thyroid abnormalities in PCOS patients and restricts comparisons with other studies where autoimmune thyroid markers were measured. The study included a relatively small sample size of 89 pregnant women; that may not be enough to cover the whole range of thyroid dysfunction in PCOS. Further multi-center studies with larger sample sizes and comprehensive thyroid assessments are essential to know the full extent of thyroid dysfunction in PCOS.

## CONCLUSIONS

Thyroid disorder was observed in about half of the cases of PCOS. High rates of menstrual irregularities, androgenic symptoms such as hirsutism and acne, and fertility issues were also prominent among PCOS patients. Other clinical manifestations were consistent with previous studies.
